# Mussel-Inspired Polydopamine as a Green, Efficient, and Stable Platform to Functionalize Bamboo Fiber with Amino-Terminated Alkyl for High Performance Poly(butylene succinate) Composites

**DOI:** 10.3390/polym10040461

**Published:** 2018-04-22

**Authors:** Gonghua Hong, Haitao Cheng, Yang Meng, Jianyong Lin, Zhenghao Chen, Shuangbao Zhang, Wei Song

**Affiliations:** 1 MOE Key Laboratory of Wooden Material Science and Application, College of Materials Science and Technology, Beijing Forestry University, Beijing 100083, China; honggonghua@bjfu.edu.cn (G.H.); lxf_mengyang@bjfu.edu.cn (Y.M.); javanlin@bjfu.edu.cn (J.L.); zhenghaochenbjfu@foxmail.com (Z.C.); 2 Department of Biomaterials, International Center for Bamboo and Rattan, Beijing 100102, China; htcheng@icbr.ac.cn

**Keywords:** bamboo fiber, mussel inspired polydopamine, fiber functionalization, polybutylene succinate, interfacial properties, biocomposite performances

## Abstract

A new and eco-friendly mussel-inspired surface modification pathway for bamboo fiber (BF) is presented in this study. The self-assembly polydopamine (PDA) coating can firmly adhere on BF surface, which also serves as a bridge to graft octadecylamine (ODA) for hydrophobic surface preparation. The as-formed PDA/ODA hybrid layer could supply abundant hydrophobic long-chain alkyls groups and generated a marked increase in BF surface roughness and a marked decrease in surface free energy. These changes provided advantages to improve fiber–matrix interfacial adhesion and wettability. Consequently, high performance was achieved by incorporating the hybrid modified BF into the polybutylene succinate (PBS) matrix. The resultant composite exhibited excellent mechanical properties, particularly tensile strength, which markedly increased by 77.2%. Meanwhile, considerable high water resistance with an absorption rate as low as 5.63% was also achieved. The gratifying macro-performance was primarily attributed to the excellent interfacial adhesion attained by hydrogen bonding and physical intertwining between the PDA/ODA coating on the BF and the PBS matrix, which was further determined by fracture morphology observations and dynamic mechanical analysis. Owing to the superior adhesive capacity of PDA, this mussel-inspired surface modification method may result in wide-ranging applications in polymer composites and be adapted to all natural fibers.

## 1. Introduction

Petroleum shortage and growing environment pollution have gradually prompted the development of biobased polymers derived from biomass materials. Owing to their superior biodegradability and biocompatibility, biobased polymers exhibit potential for application in industrial production and various aspects of life, with the aim to develop a more sustainable and eco-friendly society [1]. Among these polymers, polybutylene succinate (PBS) has been successfully synthesized by polycondensation of biofermentation monomers (1,4-butanediol and succinic acid) that can be easily obtained from natural resources, such as potato, corn, wheat, and other plants [2]. This as-synthesized biopolymer shows excellent performance, including low density, good processability, and high plasticity. These characteristics render this biopolymer applicable as a packaging, decoration, furniture, and polymeric biomedical material [3,4,5]. However, its gas-barrier properties, relatively inferior strength, and poor thermal stability at high temperature may significantly restrict further development in these aforementioned areas [6]. 

To combat these drawbacks, fiber-reinforced technique is one of the widely used methods for PBS reinforcing modification [7]. Due to the biodegradability, renewability, good availability, low cost, low density, and other characteristics, various natural fibers have attracted remarkable interest for the preparation of fully degradable polymer biocomposites [8]. Except for these attractive merits, bamboo fiber (BF) presents distinct mechanical properties with its small microfibrillar angle (2°–10°) and it is easy to obtain because of the fast growth of bamboo plants [9]. With these properties, BFs can be used as renewable fillers to reinforce all kinds of polymer matrices. Similar to other lignocellulose fibers, pristine BFs are susceptible to moisture and tend to absorb water because of the numerous free hydroxyl groups present on the surface of the fiber. Owing to this natural feature, BFs are difficult to disperse and exhibit poor wettability for low polar polymers, resulting in weak interfacial adhesion between the fiber and the matrix. This disadvantage restricts the potential of BFs as reinforcements, particularly for PBS composites [10]. In addition, the as-reinforced composite with high fiber content often exhibits low water resistance, which further renders the composite vulnerable to fungi and structural instability, markedly reducing durability and service lifetime [11]. Thus, surface modification and functionalization of BFs are critical in the fabrication of high-performance PBS composites to obtain good interfacial compatibility and anti-water capability.

Current research on surface modification of BFs have focused on decreasing surface energy, incorporating active functional groups and changing the surface structure via different treatment routes. These routes mostly include steam explosion, heat treatment, alkaline purification, acetylation, and chemical grafting with coupling agents, among others [12,13,14,15,16]. Despite the efficiency of these modified methods, the majority of the pathways are limited by a number of constraints, such as drastic processing conditions, complex synthesis steps, specific reaction devices, or severe damage to the natural structure and superior strength of the fiber itself. Moreover, disused solutions often contain massive alkali, acidic, and toxic solvents that may severely contaminate the environment. Thus, a facile, efficient and “green” functional method for the BF surface should be developed.

Fortunately, chemical catechol deposition has been rapidly developed to effortlessly decorate the surfaces of various substrates with dopamine (DA) and its derivatives by learning from the super adhesion structures (e.g., 3,4-dihydroxy-l-phenylalanine and lysine amino acids) in mussel byssus [17]. As determined by Lee et al. [18], DA, an easily accessible small-molecule compound that contains both functionalities of the aforementioned structures, can self-polymerize under particularly mild alkaline conditions, similar to the marine environment without any toxic reagents. DA can also generate a highly adhesive polydopamine (PDA) film that can firmly adhere to almost all types of as-dipped substrates, including nonstick polytetrafluoroethylene. The anchored PDA coating consisting of massive catechol groups can act as a vigorous platform for large-scale diversities of secondary functionalized reactions, including tailoring metallic nano-coatings through in-suit reducing metal ions and grafting with amino-terminated monomers or polymers via Michael addition and Schiff base reactions [19]. In recent years, many advanced functional materials have been fabricated from conventional substrates, such as fabrics, sponges, and clay minerals, via mussel-inspired surface modification [20,21,22]. Zhu et al. [23] tightly immobilized magnetic nanoparticles (Fe_3_O_4_) on a sponge surface by using PDA as an intermediate. Similarly, superhydrophobic and superoleophilic fabrics were developed by Wang et al. [24]. As expected, based on mussel-inspired chemistry, biomass resources such as BFs can be facilely decorated with catechol groups by one-step PDA coating. In addition, subsequent specific functionalization can be simply obtained on this PDA platform. Most importantly, this pathway does not damage the natural structure of BFs, which might even be protected in some cases. However, to the best of our knowledge, no previous studies have been conducted on the use of this mussel-inspired platform to further achieve the surface functionalization of BFs via the aforementioned secondary reactions to expand their applications, particularly for fiber-reinforced polymer composites.

In our pervious study [10], we found that pure polydopamine coating can act as a couple agent to improve the mechanical properties and thermal stability of BF/PBS composites. However, this modification method could not greatly reduce the water absorption of the composites. Moreover, the water resistance performance even became worse with the overloading rate of PDA particles. In the present study, we developed a new pathway to enhance interfacial adhesion between the BFs and the PBS matrix, as well as water resistance, by modifying BFs with the mussel-inspired hybrid coatings. In this study, dopamine can be physically polymerized into PDA under mild conditions (pH = 8.5) and spontaneously form an “active platform” to firmly adhere to BF surfaces. Octadecylamine (ODA), a hydrophobic long-chain amino-terminated alkyl, was then introduced onto the PDA layer via Michael addition and Schiff-based reaction. The as-modified BFs with a hybrid PDA–ODA layer was comprehensively characterized, exhibiting a rougher surface topography and lower surface free energy, compared with pristine BFs, which was more prone to easy wetting by low polar resin and form a strong interface phase. The PBS matrix was subsequently reinforced with the functionalized BFs. High performance, including excellent static and dynamic mechanical properties and superior anti-water capability, was achieved. The satisfactory results were attributed to the hybrid PDA-based layer that markedly improved the interfacial interaction between the as-deposited BFs and the PBS matrix. The outcome indicated that biomimetic surface modification exhibits potential for application in fiber-reinforced polymer composites and can be adapted to all natural fibers.

## 2. Materials and Methods 

### 2.1. Materials

BFs with a mixed particle size of 40–60 mesh (fiber length below 380 μm) was provided by Anhui Sentai WPC New Material Co. Ltd. (Huzhou, China). The PBS (crystallinity = 32%, flow index = 2.0 g/10 min and density = 1.24 g/cm^3^) used in this study as the polymer matrix was purchased from Showa Highpolymer Co. Ltd. (Tokyo, Japan). Tris (hydroxymethyl) aminomethane (Tris, 99.9% purity) and dopamine hydrochloride (assay ≥ 98.5%) were provided by Duly Biotech Co., Ltd. (Nanjing, China). Octadecylamine (ODA, AR) was obtained from Macklin Biochemical Co., Ltd. (Shanghai, China). All other reagents used in this study including diiodomethane, deionized water, anhydrous ethanol, and paraffin wax were purchased from Beijing Chemical Reagent Factory (Beijing, China). 

### 2.2. Surface Functionalization of BFs

The as-received BFs were first washed by a mixture of deionized water and anhydrous ethanol for several times to remove the impurities on the BFs surfaces, and dried in an air-dry oven at 80 °C, and were denoted as C–BFs. To obtain the PDA modified BFs, C–BFs were immersed in a buffer solution, followed by the addition of dopamine with vigorously stirring for 48 h at room temperature (RT). Such buffer solution was prepared by dissolving TRIS, which can alter pH to 8.5. After that, the nut-brown PDA coating through self-polymerization of dopamine was produced on the surface of C–BFs. The PDA-coated BFs (PDA–BFs) were obtained through centrifugation, purified with distilled water several times to remove free dopamine, and vacuum dried at 80 °C for 12 h. Next, the dried PDA–BFs were soaked into ethanol solution containing 10 mM ODA and gently stirred at RT for 12 h, followed by thoroughly rinsing with anhydrous ethanol, filtering, and drying (80 °C for 12 h). Finally, the surface functionalized BFs were prepared, which were labeled as PDA/ODA-BFs. In addition, the C-BFs were treated by only ODA without PDA coated through the same grafting route for the aim of verifying the essential function of the deposited PDA layer, and were named as ODA–BFs. The BFs based on different preparation conditions are listed in Table 1.

### 2.3. Preparation of BFs Reinforced PBS Composites

The route of the preparation of BFs/PBS composites was similar to our group’s previous research [16]. Firstly, BFs and PBS with mass ration of 1:1 were mixed in a high-speed blender at 2900 rpm for 5 min. Then, the PBS and BFs mixture was dried in an oven at 80 °C for 18 h and extruded via a co-rotating twin-screw extruder (KESUN KS-20, Kunshan, China). The temperature profile of the extruder barrel was 135/140/145/145/135 °C, and the screw speed was 180 rpm. The as-blended mixtures were carefully ground until the particle diameter was 2 mm, following by drying in an oven at 80 °C for 18 h. The composites were produced via a hot press (SYSMEN-II, China Academy of Forestry, Beijing, China) at 180 °C with a 4 MPa pressure for 6 min. The density of the composites was 1.28 g/cm^3^, and the measure was 270 × 270 × 4 mm^3^. Before demolding, the formed mat was cooled at 4 MPa in room temperature for 10 min. The formulations of biocomposites obtained from the different treated BFs are the same as those in Table 1.

### 2.4. Characterization and Measurement of BF and PBS Composites

The surface topography of the pristine and modified BFs were studied by an Atomic force microscopy (AFM, Bruker Multimode 8) using the tapping mode under a monolithic Si tip, to collect the topographic (height) and phase images of samples.

X-ray diffraction testing (XRD) was conducted in D8 Advanced Instrument (Bruker, Madison, WI, USA) to analyze the crystallization performances of the BFs samples with a Cu Kα radiation source of 1.5406 Å at the scanning angle (2*θ*) ranging from 5° to 40° with a step size of 5°/min. The crystallization index (*CI*) was obtained with Equation (1) [25]:

(1) $$CI=\left(\frac{I_{002}-I_{am}}{I_{am}}\right)\times 100\%,$$

where *I*_002_ and *I_am_* represent the peak diffractive strength of the 002 crystalline plane and non-crystalline plane, respectively.

The change of surface functional groups of the BFs samples were tested by Fourier transform infrared spectroscopy (FTIR, Vertex 70v, Bruker, Karlsruhe, Germany), the BFs samples were mixed with potassium bromide (KBr) pellets at a weight ratio of 1:100 before recording the spectra. All samples were analyzed in a wavenumber range of 500–4000 cm^−1^ under a total of 40 scans.

X-ray photoelectron spectroscopy (XPS) testing were carried out to further achieve the surface chemical/elemental states of the BFs samples in an X-ray photoelectron spectrometer (ESCALAB 250XI, Thermo Fisher Scientific Co., Ltd., Shanghai, China) at room temperature by using a monochromatic Al Kα radiation source of 1486.7 eV. All samples were acquired with a pass energy of 100 eV in increments of 0.1 eV.

Water contact angles (WCA) were examined on a DSA 100 video-based instrument (Kruss Gmbh, Hamburg, Germany). The same quality of BFs samples were first put into a tablet press (BJ-15, BOJUN science and technology Co., LTD, Tianjin, China) to form a flakelet (30 MPa, 5 min) and then 3 μL deionized water was dropped on the plane surfaces of the as-formed tablets recorded by a CCD camera at a rate of 24 frames. Besides, another non-polar liquid (i.e., diiodomethane) was used to calculate the surface free energy of the samples. Every sample was measured at ten different regions and the wettability specification of the above mentioned two liquid are shown in Table 2.

The static mechanical properties of the BFs/PBS composite specimens were measured using an electronic universal testing machine (Kexin Instrument Equipment Co. Ltd., Changchun, China) and a cantilever beam impact tester (Hebe Precision Testing Machine Co. Ltd., Chengde, China). The tensile, flexural, and impact properties were tested based on the standards of ASTM D638-03, ASTM D790-03, and ASTM D256-03, respectively. Firstly, all specimens were cut and profiled in specific sizes with dimensions of 80 mm × 13 mm × 4 mm for flexural test, 80 mm × 10 mm × 4 mm for impact test, and 160 mm × 20 mm × 4 mm (dumbbell-shape, with a radius of arc of 76 mm, gauge length of 50 mm, and middle part width of 13 mm) for tensile test. The flexural test was carried out with a speed of 10 mm/min under the three-point bending model, and the span between the two fulcrums was 64 mm. The tensile property was measured with a testing speed of 2 mm/min. The impact test was conducted with an impact energy of 2 J, and the moment of force was 1.7717 Nm. Eight replicates were chosen in each test groups, and the final results were determined by calculating the average values.

The dynamic mechanical properties of the composites samples were conducted on Dynamic Thermomechanical Analyzer (DMA, NETZSCH, Selb, Germany). The samples with dimensions of 35 mm × 10 mm × 3 mm were measured at the scanning frequency of 1 Hz, and the heating rate of 3 °C/min. The test temperature ranged from −80 to 100 °C. All of the samples were tested three times to avoid errors.

The water absorption capability of the PBS composites were conducted according to ASTM D5229-12. The samples with dimensions of 10 mm × 10 mm × 4 mm were first over-dried at 80 °C to achieve the absolutely dry weight (*W_d_*), and then immersed into a deionized water beaker at the temperature of 25° ± 1 °C for 13 days. After removal of the remaining water on surfaces, the samples were weighted at intervals of 12 h. Each group contained five specimens and the resultant water absorption rate (*M*) was achieved using Equation (2):

(2) $$M=\left(\frac{W_{t}-W_{d}}{W_{d}}\right)\times 100\%,$$

where *W_d_* and *W_t_* represents the average values of the absolutely dry weight and the instantaneous weight at the intervals, respectively. Fick’s law (*M_t_*/*M*_e_ = *kt^n^*) was used to achieve the absorption kinetic performance of the composites through parameters fitted based on Equation (3) [27]:

(3) $$\log\;\left(\frac{M_{t}}{M_{e}}\right)=\log\;(k)+n\log\;(t),$$

where *M_t_* and *M*_e_ represent the water absorption rate at time *t*, and the equilibrium moisture state, respectively.

The interface adhesion properties of PBS composites were investigated on a FEI Quanta FEG 650 scanning electron microscopy (SEM, Thermo Fisher Scientific Co., Ltd., Waltham, MA, USA) to observe the fracture morphology that was obtained from the tension-fractured samples, at an operation of 10 kV. Before observation, Au layer (8–10 nm) was coated on the specimen surface.

## 3. Results and Discussion

### 3.1. Reaction Mechanism of Depositing Mussel-Inspired Functional Coatings on Bamboo Fibers

**Scheme 1:**
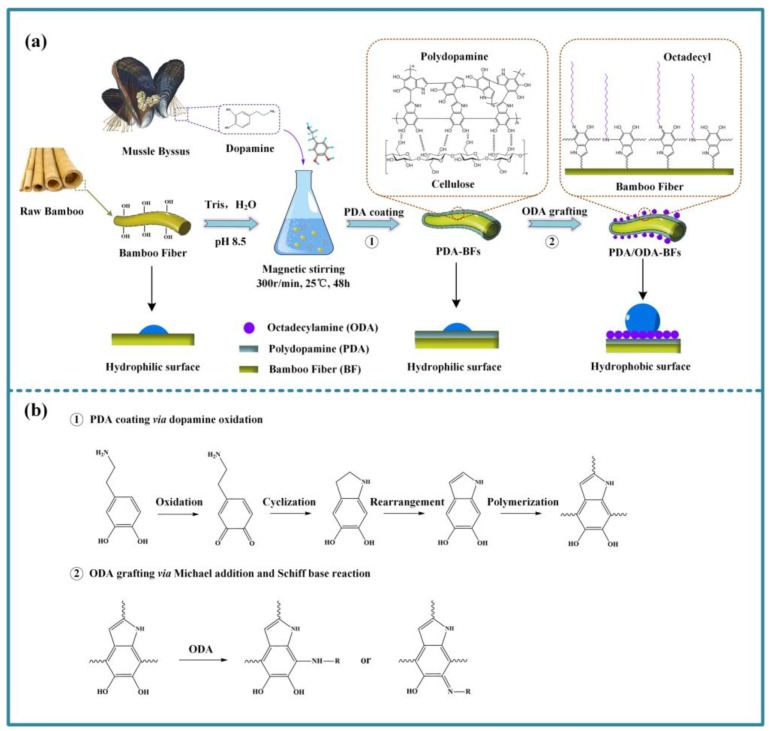
The reaction mechanism of depositing mussel-inspired functional coatings on pristine bamboo fibers (BFs). (**a**) Surface modification process of BFs; and (**b**) chemical reaction mechanism: ① illustration of oxidation self-polymerization of dopamine [22]; and ② potential mechanism of grafting long-chain alkylamine (octadecylamine) onto as-prepared PDA coating to acquire hydrophobic properties via Michael addition and Schiff based reactions.

A detailed procedure of establishing bionic hydrophobic structures on BF surfaces is presented in Scheme 1. Bamboo-derived materials (BDMs) can be simply ornamented with catechol groups when soaked into a weak alkaline dopamine (DA) aqueous solution at ambient temperature. In this environment, PDA was assembled via oxidation self-polymerization of DA and tightly adhered to the BDM surfaces by hydrogen bonds, forming a robust catechol functional film, as verified by Peng et al. [28]. The surface polarity of BFs can hardly be altered merely by primary mussel-inspired surface modification because of the presence of numerous hydrophilic groups, such as hydroxyl, carbonyl, and amino groups, on PDA molecules. Subsequent reaction to graft NH-terminated octadecyl (octadecylamine, ODA) on PDA films via Michael addition and Schiff-based reactions can introduce anti-water groups into BFs, which was a critical factor for building hydrophobic surfaces [9]. This long-chain alkylamine can also create hierarchical rough structures on BF surfaces, which can further enhance water resistance, as in lotus leaves [22]. Thus, hydrophobic BFs were eventually created.

Chen et al. improved the water resistance of lignocellulose fibers by high-temperature hot air and silane coupling co-modification: the number of free hydroxyl groups on fiber surfaces showed a decreasing tendency with an increase in the temperature of hot air [29]. Zhang et al. reduced the surface polarity of BFs via alkali treatment with sodium hydroxide [14]. However, the harsh reaction environment, including hot air and strong alkaline solution, destroyed the natural structures and components of lignocellulose fibers. In the current study, bionic multifunctional surface modification was conducted under mild conditions, which led to the hydrophobicity of the BF surface while retaining its distinct structure and components (Figure S1). The anti-water surfaces improved the dispersion of BFs in the polymer matrix; meanwhile, the interfacial bonding strength was enhanced by twisting the polymer molecular chain together with long-chain octadecyl groups. The ultimate biocomposites represented excellent mechanical performance attributed to the undamaged BFs.

### 3.2. Surface Topography of Bamboo Fibers

**Figure 1:**
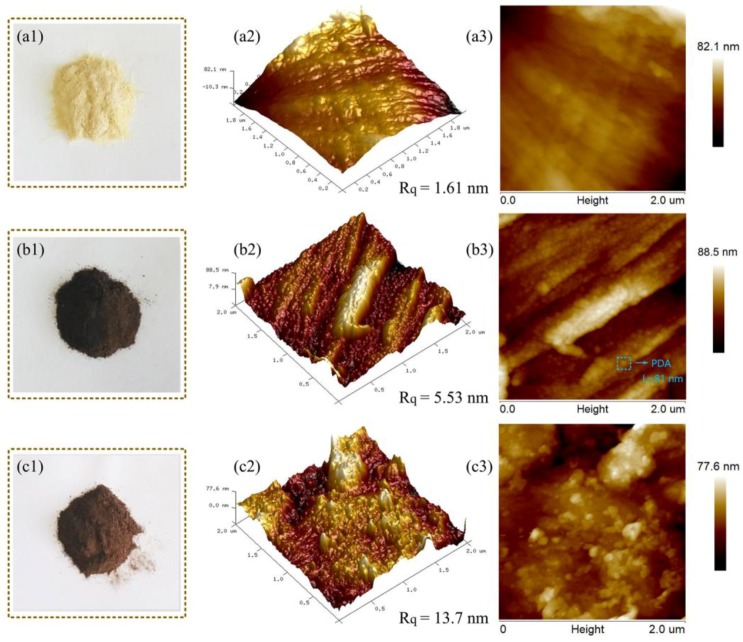
Optical photographs, atomic force microscopes (AFM) 3D and 2D height images of: (**a1**–**a3**) C–BFs; (**b1**–**b3**) PDA–BFs; and (**c1**–**c3**) PDA/ODA–BFs.

Figure 1 shows the optical photographs and AFM 3D and 2D height images of C–BFs, PDA–BFs, and PDA/ODA–BFs. The pristine BFs presented a light-yellow color (Figure 1a1) and exhibited a relatively smooth surface at the nanoscale with a root–mean–square roughness (*R*_q_) of 1.61 nm (Figure 1a2,a3). After PDA deposition, the color of the PDA–BFs changed to dark gray because of the oxidative self-assembly of DA on the BF surfaces (Figure 1b1). The as-formed PDA particles showed a nanosphere structure with a scale of around 81 nm. The particles were uniformly dispersed on the BF surfaces, exhibiting a spherical apophysis nanonet morphology. *R*_q_ increased from 1.61 nm to 5.53 nm, as shown in Figure 1b2,b3. The secondary reaction to grafting ODA onto the catechol functional layer caused the color of the PDA/ODA–BFs to become slightly lighter than that of the PDA–BFs (Figure 1c1). With the incorporation of long-chain alkyl groups, the surface topography of PDA/ODA–BFs that exhibited a complex and uneven microstructure was markedly different from the two aforementioned BFs, forming a hybrid-coated rough surface with *R*_q_ = 13.7 nm. These results indicate that the anti-water hierarchical structure was successfully deposited on the BF surface via mussel-inspired polydopamine modification. In addition, the surface area of BFs can be improved because of the increase in surface roughness, which can potentially influence mechanical interlocking between BFs and the polymer matrix, similar to the silane coupling agent [30]. 

### 3.3. Microstructural and Surface Chemical Composition Analysis of Bamboo Fibers

**Figure 2:**
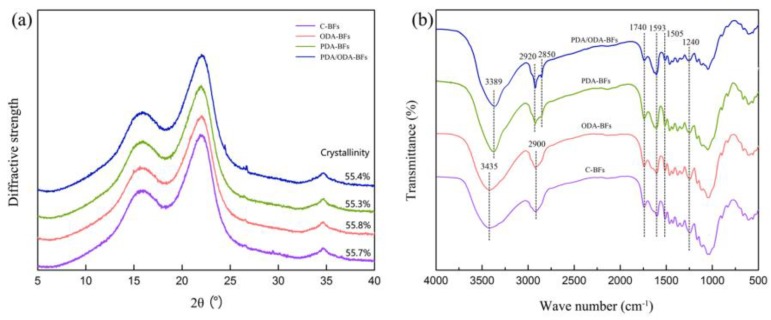
XRD patterns (**a**); and FTIR spectrum (**b**) for the C–BFs, ODA–BFs, PDA–BFs, and PDA/ODA–BFs samples.

XRD was conducted to investigate the crystallinity of BFs. The XRD patterns of pristine BFs (C–BFs) and surface-modified BFs are shown in Figure 2a. The C–BFs exhibited typical XRD patterns of lignocellulose materials, to which were attributed the (101), (002), and (040) characteristic diffraction peaks of native cellulose I [25], located at the 2θ angles of 17°, 22.5°, and 35°, respectively. The subsequent modification process of coating PDA and grafting ODA onto the BF surfaces exerted no apparent effects on the typical XRD patterns of the BFs with respect to the shapes and 2θ angles. These results indicate that mussel-inspired modification can hardly penetrate the inner molecular layers of the cellulose and damage the structure of crystal zones in the cellulose chain, preserving the high crystallinity of natural BFs within the 55.3% to 55.8% range.

The FTIR spectra of the C–BFs, ODA–BFs, PDA–BFs, and PDA/ODA–BFs were investigated to determine the chemical groups of the pristine and surface-modified BFs, as shown in Figure 2b. For the untreated (C–BFs) samples, a strong absorption peak located at 3435 cm^−1^ was assigned to the stretching vibration of hydroxyl (–OH) groups, and a peak at 2900 cm^−1^ was designated as C–H stretching vibrations [16]. As for the spectra in the fingerprint zone ranging from 2000 cm^−1^ to 500 cm^−1^, the peaks at 1740 cm^−1^ represented the stretching vibration of carbonyl (C=O) groups. The stretching vibration of acyl-oxygen (CO–OR)/benzene–oxygen groups was observed at 1240 cm^−1^ [15]. The spectral bands at 1593 and 1505 cm^−1^ corresponded to the carbon skeleton vibration of the benzene ring [9]. These four peaks characterized the main components of biomass materials derived from bamboo including cellulose, hemicellulose, and lignin. This finding was consistent with the FTIR spectra investigated by Zhang et al. [14]. After treatment with PDA (PDA–BFs), the bond at 3435 cm^−1^ was offset to 3389 cm^−1^, which was attributable to the hydrogen bond between PDA molecules and bamboo hydroxyl groups [19]. According to previous research [24,31], the characteristic peaks of PDA located at 1510, 1600, and 1274 cm^−1^ were designated as N–H shearing vibration, stretching vibration from the indole ring, and C–O stretching vibration from phenolic moieties, respectively. However, the spectra of modified BFs provided no apparent proofs of the presence of PDA. The reason might be that bamboo is an intricate natural polymer composed of lignin and polyphenolic extracts, which may present many similar functional groups with polydopamine. Notably, the two characteristic peaks at 2920 and 2850 cm^−1^ for the PDA–BF and PDA/ODA–BF samples were attributed to –CH_3_ and –CH_2_ asymmetrical stretching vibrations and symmetric stretching vibrations from the PDA molecules, respectively [22], which proved that PDA-coating was successfully conducted. Moreover, in the PDA/ODA–BF spectrum, the doublets at 2920 and 2850 cm^−1^ were extremely high. The C–H stretching vibration in long-chain alkyl of ODA led to this occurrence because of the numerous methyl and methylene groups in ODA chains. This finding indicates the successful grafting of ODA onto the PDA–BF samples. The location and intensity of the characteristic peaks in the ODA–BF samples were almost identical to those in the untreated samples, indicating that the chain alkyl amine cannot be grafted to the surface of the BFs without PDA as a reaction platform.

**Figure 3:**
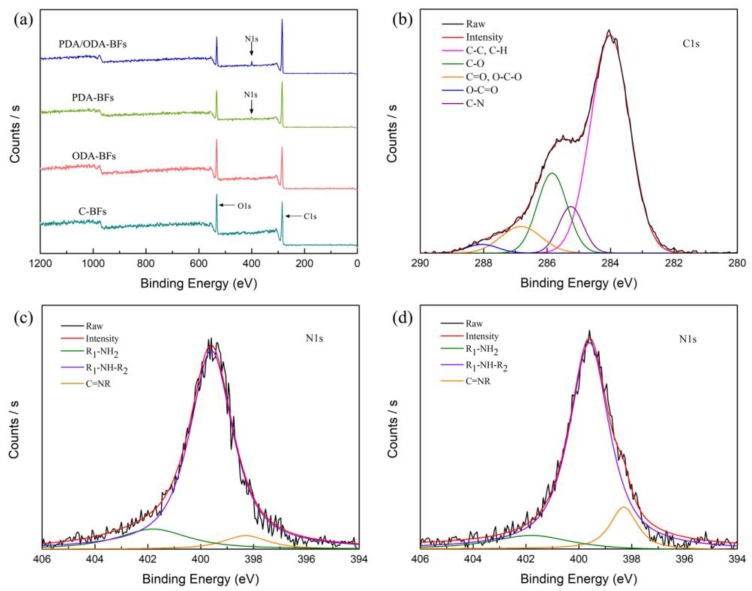
XPS wide-scan and high resolution spectra of BFs samples: (**a**) wide-scan XPS spectra of C–BFs, PDA–BFs, ODA–BFs and PDA/ODA–BFs; (**b**) C 1s core-level of PDA–BFs; (**c**) N 1s core-level of PDA–BFs; and (**d**) N 1s core-level of PDA/ODA–BFs.

To elucidate the surface chemical/elemental states of the BF samples during modification, XPS analysis was conducted, as presented in Figure 3 and Table 3. As shown in Figure 3a, the peaks of C1s (284 eV) and O1s (532 eV) were observed in all samples, similar to those that can be observed in other natural lignocellulose materials [9]. A new signal of N from PDA molecules with 6.23% content emerged in the spectrum of PDA–BFs, demonstrating that the PDA layers were successfully ornamented on the BF surfaces. The atom content (At. %) of carbon (77.75%) was higher and the N/C atom ratio (0.071) was lower in the PDA/ODA–BFs surfaces than in the PDA–BF surfaces. These differences were attributed to the long-chain octadecyl grafted onto the PDA-coated BFs. Figure 3b exhibits the C 1s high resolution spectra of PDA–BFs, which could be peak-fitted into five segments. The characteristic peaks of the binding energy (BE) at 284.0 eV for the C–C and C–H species, 286.0 eV for the C–O species, 286.8 eV for the C=O and O–C–O species, and 288.5 eV for the O–C=O species were commonly observed on the other bamboo materials [32]. Interestingly, an additional peak appeared with BE located at 285.5 eV, which was ascribed to the C–N species from the as-coated PDA molecule [22]. Figure 3c,d presents the N 1s high resolution spectral lines of the PDA–BFs and PDA/ODA–BFs. The core-level spectra of these two samples can be peaked-fitted into three segments, including the R–NH_2_ species (primary amine, 401.8 eV), R_1_–NH–R_2_ species (secondary amine, 398.6 eV), and C=NR species (tertiary or aromatic amine, 398.6 eV), respectively [33]. As for the PDA/ODA–BFs, all intensities of the secondary amine and tertiary/aromatic amine species were enhanced, with the contents increasing from 79.68% to 81.08% and 6.72% to 11.47%, respectively. These increases may be attributed to ODA grafting onto the PDA–BFs surfaces via Michael addition or Schiff-base reactions, which formed two different types of resultant products, as demonstrated in Scheme 1. These findings, combined with the results of FTIR analysis, clearly indicate that PDA and ODA coatings were successfully introduced on the BF surfaces to obtain hydrophobic properties. However, the signals of the ODA–BFs samples were similar to those of the pristine BFs, which confirmed that the long-chain alkyl amine can hardly graft onto the surface of the BFs without PDA coating as a reaction platform.

### 3.4. Surface Wettability of Bamboo Fibers

**Figure 4:**
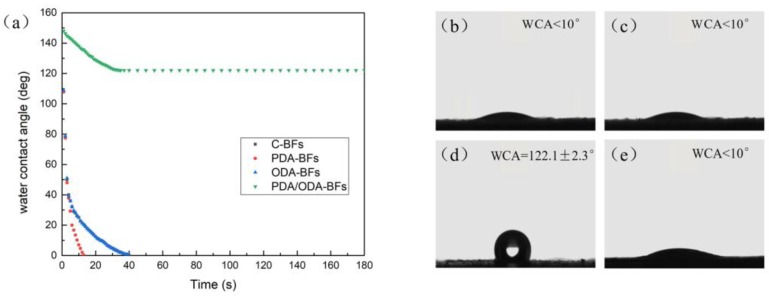
Hydrophobic performance characterization: (**a**) water contact angles as a function of time for BFs samples; and micrographs of 3 μL deionized water droplets on the plane surfaces of: (**b**) C–BFs; (**c**) PDA–BFs; (**d**) PDA/ODA–BFs; and (**e**) ODA–BFs.

Figure 4a demonstrates the changes in water contact angles (WCAs) on the surfaces of the BF samples over time. The WCA on the C–BFs surface decreased rapidly over a short period of 40 s. This reduction could be attributed to the existence of many free hydroxyl groups on the BF surfaces, which exhibited affinity for water molecules. Meanwhile, the WCA on the PDA–BFs decreased more rapidly, i.e., in 20 s. This rapid reduction was attributed to the coating of PDA on the BFs, introducing more hydrophilic groups (e.g., –OH, –NH) that promoted such change. Notably, the long-chain alkyl grafts on the PDA layers changed the surface polarity of the natural BFs, with the WCA decreasing rapidly in 40 s and then reaching the equilibrium above 120°, which showed hydrophobic properties. However, the change in the WCA on the ODA–BFs was not as apparent as that in the C–BFs, suggesting that the hydrophobic ODA coating could hardly graft onto the BFs without as-coated PDA layers. These occurrences could be observed in micrographs recorded with a CCD camera. When dripped on BFs tablets, the water drops soaked into the hydrophilic tablets (separate samples of C–BFs, PDA–BFs, and ODA–BFs) with a WCA below 10° (Figure 4b,c,e); meanwhile, the drops remained on the surface of hydrophobic tablet (PDA/ODA–BFs) with a WCA of 122.1° ± 2.3° (Figure 4d).

The wettability of the BFs exerts a critical effect on the interfacial interaction between the fibers and the polymer matrix. Reinforcements with lower surface energy promotes the wettability of the as-reinforced low polar polymer and significantly enhances the interfacial properties [27]. The surface free energies of the BF samples were calculated according to Young’s equation, as shown in Equation (4) [26]:

(4) $$\gamma_{S}=\gamma_{L}\cos\theta \;+\;\gamma_{SL},$$

where *γ_S_*, *γ_L_*, and *γ_SL_* represent the surface tension of the solid, liquid, and solid–liquid interfaces, respectively. *θ* represents the initial contact angle between the solid (*S*) and the liquid (*L*) interfaces. The OWRK method (Note S1 in Supplementary Materials) [34] was also used to determine the value of *γ_S_*, as presented in Equation (5):

(5) $$\gamma_{L}\left(1+\cos\;\theta \right)=2\sqrt{\gamma_{S}^{d}\gamma_{L}^{d}}+2\sqrt{\gamma_{S}^{p}\gamma_{L}^{p}},$$

where $\gamma_{S}^{d}$ and $\gamma_{L}^{d}$ represent the dispersion components on the surface of the solid and liquid interfaces, respectively. $\gamma_{S}^{p}$ and $\gamma_{L}^{p}$ denote the polar components on the surface of the solid and liquid interfaces, respectively. The values (*p*-values < 0.05) are listed in Table 4. C–BFs exhibited a high surface free energy (*γ_S_*) of 45.57 mJ/m^2^, which was consistent with the reported findings ranging from 40 mJ/m^2^ to 60 mJ/m^2^ [35]. Based on Young’s equation, the surface free energy *γ_S_* could be divided into two components, namely, the dispersive component $\gamma_{S}^{d}$, which was susceptible to the topography of the BFs, and the polar component $\gamma_{S}^{p}$ dominated by the active chemical groups on the BFs [36]. After PDA modification, the polar component $\gamma_{S}^{p}$ increased from 7.03 mJ/m^2^ to 12.68 mJ/m^2^, which was contributed by the additional hydrophilic groups provided by PDA, resulting in a higher surface free energy of 49.96 mJ/m^2^. However, the surface free energy of the PDA/ODA–BFs markedly decreased to 34.45 mJ/m^2^. This reduction was attributed to both the polar and dispersive components. The introduction of the hydrophobic ODA to the surface of the PDA–BFs markedly reduced the polar component $\gamma_{S}^{p}$ from 12.68 mJ/m^2^ to 1.46 mJ/m^2^. Meanwhile, the hierarchical rough structures established on the BF surfaces, as shown in Figure 1, led to the decrease in the dispersive component $\gamma_{S}^{d}$ from 37.28 mJ/m^2^ to 32.99 mJ/m^2^. In addition, the ratio of $\gamma_{S}^{d}$ to $\gamma_{S}^{p}$ was a crucial index for evaluating the wettability of the substrates. Interestingly, the highest ratio of 22.60 was observed in the PDA/ODA–BF samples, indicating that this final as-modified BFs exhibited a hydrophobic and oleophilic surface. Consequently, this outstanding surface with a lower surface energy indicated a considerable affinity for as-reinforced low polar polymers and a strong interfacial bonding between BFs and the low polar resin matrix was expected to be obtained.

### 3.5. Static Mechanical Properties of BF/PBS Composites

As discussed earlier, the adhered ODA–PDA layer introduced a massive hydrophobic group onto the BF surfaces, decreasing the surface free energy and rendering the BFs more facilely to be wetted by low polar polymers. Subsequently, the effectiveness of this bionic functionalization strategy was investigated by evaluating the macro and micro performances of the BF-reinforced PBS composites.

Table 5 lists the static mechanical properties of the PBS-based composites. Relative to those of the control groups prepared with pristine BFs (C–BFs), the tensile strength, tensile modulus, flexural strength, and flexural modulus of the PDA–BFs/PBS composites increased by 14.4% from 13.62 MPa to 15.58 MPa, 5.3% from 0.75 GPa to 0.79 GPa, 7.5% from 31.38 MPa to 33.72 MPa, and 5.4% from 2.05 GPa to 2.16 GPa, respectively. These increases, which might be attributed to the improved surface roughness by PDA coating, as presented in Figure 1, led to an increase in specific surface area, which increased the physical bonding area between the PBS matrix and the reinforcements. By contrast, the as-coated PDA layer also acted as a rigidity agent that decreased the plasticity of the PBS matrix [37], which led to a slight reduction in impact strength from 10.25 kJ/m^2^ to 9.02 kJ/m^2^. However, the enhancement of the mechanical properties resulting from the pure PDA coating was limited. Owing to the hydrophilic nature of PDA molecules, the improvement of the mechanical properties of the PBS composites was not mainly caused by the increase in the surface roughness of the PDA–BFs. As expected, the groups with PDA–ODA modification (PDA/ODA–BFs) showed the best mechanical performances. The as-coated PDA–ODA layer contributed an improvement of 77.2% (from 13.62 MPa to 24.13 MPa) in tensile strength as well as 35.3% (from 0.75 GPa to 1.16 GPa) in tensile modulus, compared with the C–BFs. Similarly, the flexural strength and modulus were increased to 40.5% (from 31.38 GPa to 44.09 GPa) and 30.2% (from 2.05 GPa to 2.67 GPa), respectively. These results could result from the outstanding wettability and impressive topography of the PDA/ODA–BFs surfaces, which allowed the uniform dispersion of BFs in PBS. Meanwhile, the interfacial bonding of the composites was enhanced, leading to an effective transfer of stress between the fiber and the matrix. Good interfacial compatibility was reported to hamper energy propagation in the PBS matrix and form partial stress, causing the composites to become brittle with a lower impact strength [38]. An unexpected result was observed in the PBS composites reinforced with PDA/ODA–BFs; the impact strength increased by 32.4% from 10.25 kJ/m^2^ to 15.16 kJ/m^2^, relative to that of C–BFs. Owing to hydrophilicity, the pristine BFs easily agglomerated, generating high-stress concentration zones in the composites. The good dispersion of the PDA/ODA–BFs in PBS could divide these high-stress zones into numerous small ones, resulting in a homogeneous distribution of impact energy. In addition, impact energy could also be absorbed by the additional organic molecules (i.e., long-chain octadecyl) on the interfacial layer [39]. The increment in the impact strength of the composites reinforced with PDA/ODA–BFs is thus elucidated.

### 3.6. Dynamic Mechanical Analysis of BF/PBS Composites

Based on the viscoelasticity of the PBS matrix, dynamic mechanical analysis (DMA) was conducted to effectively evaluate the interfacial property of the as-reinforced PBS composites [40]. Three essential parameters can be obtained under dynamic loading with periodic oscillations and increased temperature. Storage modulus (*E*’) represents the capacity of energy stored in the materials, which is fairly sensitive to the stiffness behavior of composites; loss modulus (*E*’’) is related to the dissipation of energy in the polymers; tan *δ*, namely the loss factor, is the ratio of E’ to E’’, which represents the degree of polymer molecular mobility in composite materials. 

**Figure 5:**
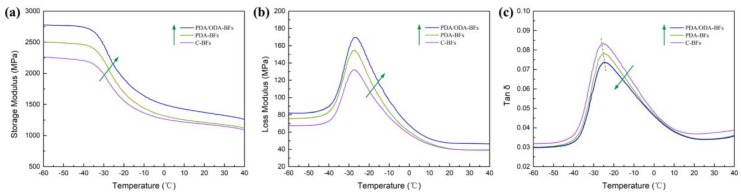
DMA curves of PBS composites: (**a**) storage modulus; (**b**) loss modulus; and (**c**) loss factor (tan *δ*).

Figure 5 exhibits the DMA curves of the PBS composites. Figure 5a shows that the storage moduli of all samples present a sharply decreasing trend with an increase in temperature from −60 °C to 40 °C, which is in accordance with the typical characteristics of thermoplastic polymers [41]. The introduction of PDA coatings to the BF surfaces increased the *E*’ of the pristine BF/PBS composites because of the toughness of PDA nanoparticles on the matrix [37]. The *E*’ of the composite was further improved by grafting ODA on the PDA layer (PDA/ODA–BFs). This finding indicates that the interfacial adhesion between the fibers and the matrix was enhanced, which was consistent with the static flexural testing results. Enhanced interference can generally restrict the PBS molecular movement, leading to an increase in viscosity, which also caused the higher loss modulus *E*’’ [40]. As shown in Figure 5b, the *E*’’ of the samples exhibited an upward trend within the temperature range after the primary modification with PDA and the subsequent reaction with ODA. Notably, the highest *E*’’ was obtained in the hybrid PBS composites modified with PDA–ODA (PDA/ODA–BFs). The loss factor (tan *δ*) as a function of temperature was calculated, as presented in Figure 5c. Damping in the glass transition zone represented the dissipation of energy for the irreversible intermolecular movement and the deformation within the polymers. The *T*_g_ (i.e., the peak temperature) value of the modified composites increased relative to that of the pristine BF/PBS composites. Similarly, the PDA/ODA–BFs/PBS composites exhibited the lowest peak intensity of tan *δ* as well as the peak area. This result demonstrates that molecular mobility was lowest in the BF/PBS composite modified by hybrid PDA–ODA, exhibiting the least energy consumption to overcome the interfriction generated by the sliding of molecular chains on the interface, which may be attributed to the favorable interfacial interaction between the PDA/ODA–BFs and the PBS matrix. Consequently, the DMA result was consistent with the aforementioned static mechanical properties and again verified the enhancement effect of the hybrid PDA–ODA coating for the BF/PBS composites.

### 3.7. Water Absorption Capability of BF/PBS Composites

**Figure 6:**
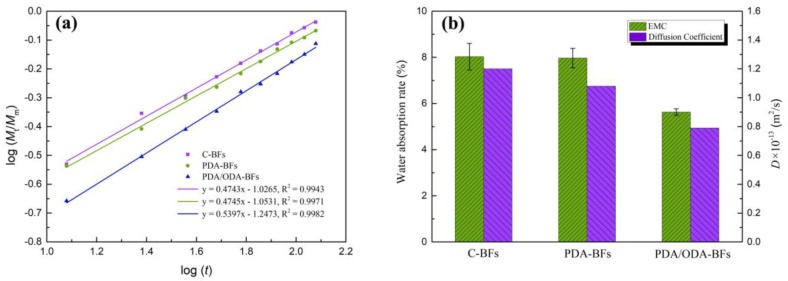
Effects of fiber surface modification on: (**a**) diffusion case fitting plots for BFs/PBS composites; and (**b**) equilibrium moisture content and diffusion coefficient of the composites.

The water uptake of all samples (*p*-values < 0.05) increased distinctly within the first 120 h and then showed a decreasing trend, ultimately reaching equilibrium, as shown in Figure S2. To further obtain the kinetics of water uptake behavior in the composites, the experimental data was linearly fitted according to Fick’s laws (Equation (3)). Figure 6a shows that the diffusion cases are in accordance with Fick’s laws (*R*^2^ > 0.98) with *n* values close to 0.5 (Table S1), indicating that the water uptake of all samples can be categorized as Fickian behavior [27]. In addition, to better analyze the velocity of the water molecules that infiltrated into the composites under Fickian behavior, the diffusion coefficient (*D*) was determined based on Equation (6) [9]:

(6) $$D=\pi {\left[\left(kh\right)/\left(4M_{e}\right)\right]}^{2},$$

where *k* is the kinetics parameter in Equation (4), and *h* is the initial thickness of the samples prior to immersion. The equilibrium moisture content (*M*_e_) and calculated *D* values of the BF/PBS composites with different treatments are shown in Figure 6b (the data are listed in Table S1). PBS can only absorb about 1% of water because of hydrophobicity [42]; thus, the dominant factor to influence the WA of the composites was the hydrophilicity of the natural fiber and the interfacial bonding between the fiber and the PBS matrix. The unmodified composites (C–BFs) exhibited the highest *k* and *D* values, as well as the equilibrium moisture content (*M*_e_) of up to 8.03%, which could be attributed to the existence of massive free hydroxyl groups on the surface of the BFs with a strong affinity for water molecules. In addition, the wettability between the PBS matrix and the unmodified BFs were not desirable, resulting in poor interface bonding, which allowed the water molecules to easily penetrate the gap between the composites. Compared with the control groups, the primary PDA coating could hardly improve the water resistance of the composites (PDA–BFs), with *M*_e_ only decreasing from 8.03% to 7.97%. This finding could be attributed to the introduction of new hydrophilic groups (–OH, –NH) from PDA molecules that once again promoted the WA of the BFs. Meanwhile, the pure PDA coating enhanced the interfacial bonding because of the increase in the surface roughness of the BFs, causing this nonsignificant improvement. Interestingly, the water uptake of the PDA/ODA–BFs/PBS composites considerably decreased with the lowest *M*_e_ of 5.63%. The significantly reduced *k* demonstrated the incompatible performance of the composites with water molecules, which could prevent the infiltration of water into the composites, as indicated by the reduced *D* values. This occurrence could be due to the hydrophobic long-chain alkyl groups grafted onto the as-coated PDA layers that changed the surface polarity of the BFs with hydrophobicity. Moreover, the enhanced fiber/polymer matrix interface could alleviate water infiltration into the BFs through the voids between the matrix and the fibers, which led to the good water resistance of the final composite (PDA/ODA–BFs). These results were also consistent with the aforementioned analysis of the surface wettability of the BFs (Section 3.4).

### 3.8. Fracture morphology of BF/PBS composites

**Figure 7:**
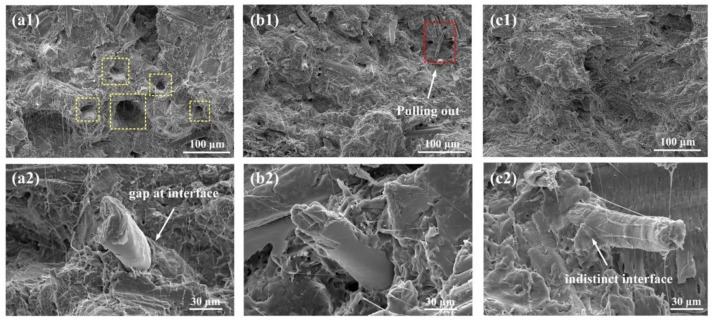
SEM fracture micrographs of: (**a1**,**a2**) C–BFs/PBS; (**b1**,**b2**) PDA–BFs/PBS; and (**c1**,**c2**) PDA/ODA–BFs/PBS composites at different magnifications.

An excellent interface phase was formed between the hybrid PDA–ODA modified BFs and the PBS matrix, resulting in superior mechanical properties and anti-water behaviors, which were also determined by SEM observation. The tensile-fractured cross-section micrographs of the PBS-based composites are shown in Figure 7.

In the C–BFs/PBS composites (Figure 7a1), many irregular holes (marked with yellow square) with diameters larger than that of a single fiber could be clearly observed in the fractured cross-section because of the pull-out of agglomerate BFs from the PBS matrix under external tension loading. The surface of the BFs that remained on the fracture surface (Figure 7a2) was smooth and hardly adhered by the PBS resin. In addition, the gap at the interface between the BF and the PBS matrix was apparently visible, demonstrating that the unmodified BF was debonded with the PBS matrix. This occurrence could be attributed to the presence of massive free hydroxyl groups on the surface of the C–BFs, which allowed these fillers to easily agglomerate together and poorly disperse in the matrix, thereby weakening the interfacial adhesion between the two parts. Debonding and failure were likely to occur in this weak interfacial zone. When the PBS resin was reinforced by the PDA–BFs, the large holes disappeared, and only few small voids remained on the fracture surface (Figure 7b1). The gap remained at the interface between the BF and PBS matrix. As shown in Figure 7b2, the pulled-out single fiber was partially adhered by PBS and presented a relatively rougher surface with the remaining PBS fragments, indicating the difficulty of forming a strong interphase in the composites by the mere introduction of pure PDA layers. As expected, a perfect fractured cross-section was observed in the PDA/ODA–BFs/PBS composites. In this case, most BFs were completely implanted in the PBS matrix and not pulled out almost entirely; thus, holes hardly appear (Figure 7c1). In addition, the single fiber was well wrapped in the PBS matrix, and a tightly integrated interface was formed between the fiber and the matrix with a boundary that was difficult to distinguish (Figure 7c2). This observation proved the strong bonding enhanced by the grafted hydrophobic and oleophilic long-chain ODA molecules. The dispersion of BFs was also remarkably homogeneous in the matrix, which again verified that the enhanced compatibility between the PDA/ODA–BFs and the PBS resin. Such improved interfacial adhesion would definitely lead to superior anti-water behaviors and superior mechanical performance, as described earlier.

### 3.9. Interfacial Adhesion Mechanism

**Scheme 2:**
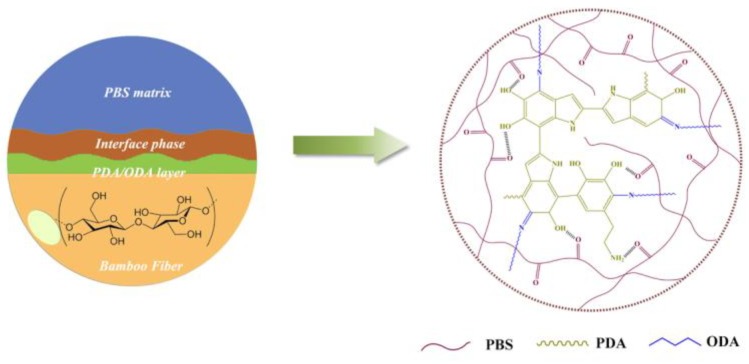
The proposed schematic of interfacial interaction in the interface phase of PDA/ODA–BFs/PBS composite.

Based on the aforementioned characterizations, we tentatively proposed the mechanism of as-enhanced interfacial adhesion achieved by establishing the PDA–ODA layer on the BF surface in Scheme 2. The hybrid PDA-based functional layer was facilely decorated onto the BF surface by a simple primary dip-coating method via oxidation self-polymerization of dopamine and secondary grafting NH-terminated octadecyl via Michael addition and Schiff-based reaction. This technique introduced massive active catechol groups and hydrophobic–oleophilic long-chain ODA in the fiber, as discussed in Scheme 1. The rich functional groups of long-chain octadecyl markedly decreased the surface free energy of BFs and facilitated surface wetting by the low polar PBS matrix. Meanwhile, the PBS chains are highly inclined to be intertwined with these long-chain groups, leading to an improvement in the interfacial adhesion of the BF/PBS composites. In addition, the primary adherent PDA layers also contained numerous unreacted small functional groups, including phenolic hydroxyl (–OH) and primary or secondary amine groups (–NH_2_ and –NH–), which could form hydrogen bonding with ester groups of PBS chains and once again enhance the interfacial adhesion, as confirmed by Zhou [43]. Consequently, combined with the non-covalent force and physical intertwining, a remarkable interface phase was ultimately generated to tightly adhere the BFs to the PBS resin, allowing for the uniform transfer of stress from the matrix to the fibers and preventing water from penetrating the reinforcements, thereby improving the macro performance significantly.

## 4. Conclusions

Prompted by highly adhesive structures in mussel byssus, a facile and eco-friendly PDA-based modification approach was developed to establish a hydrophobic and oleophilic surface on BFs to improve the interfacial adhesion of BF/PBS composites. The hybrid PDA-based layers were successfully coated on the BF surfaces by primary oxidation self-polymerization of dopamine and secondary grafting ODA via Michael addition and Schiff-based reaction, and almost no damage was observed in the natural structure of the as-adhered fibers, as confirmed by AFM, XPS, FTIR, and XRD. In addition, the as-formed PDA–ODA coatings decreased the surface free energy of the BF surfaces and promoted the wettability between the fibers and the as-reinforced low polar PBS matrix. Consequently, the mechanical properties of the PDA/ODA–BFs/PBS composites were markedly improved. The final flexural strength, flexural modulus, tensile strength, tensile modulus, and impact strength were increased by 40.5%, 30.2%, 77.2%, 35.3%, and 32.4%, respectively. The loss and storage moduli and water resistance capability of the PDA/ODA–BFs/PBS composites also markedly improved. The decrease in loss factor as well as the increase in glass transition temperature revealed the impressive interfacial interaction between the fillers and the PBS matrix, which was further determined by SEM observations of the fracture morphology. The significantly strengthened interfacial performances were largely attributed to the existence of the PDA–ODA hybrid layer between the resin and the reinforcements, which could bridge with PBS in the matrix by physical intertwining and hydrogen bonding, resulting in considerably improved bulk performance. Briefly stated, this green and simple mussel-inspired surface modification pathway can be potentially used to improve the interfacial strength of various BF-reinforced composites and be adapted to all natural fibers.

## Figures and Tables

**Table 1:** Surface Modification of BFs and Formulations of Biocomposites under Different Conditions.

No.1	Labels	DA (mg/mL)	ODA (mM)	BFs (wt %)	PBS (wt %)	Paraffin wax (wt %)
1	C–BFs	-	-	49	49	2
2	PDA–BFs	2	-	49	49	2
3	PDA/ODA–BFs	2	10	49	49	2
4	ODA–BFs	-	10	49	49	2

**Table 2:** Surface Tension and Components of the Test Liquids [26].

Test liquids	Surface free energy (mJ/m^2^)				
	$\gamma_{L}$	$\gamma_{L}^{LW}$($\gamma_{L}^{d}$)	$\gamma_{L}^{AB}$($\gamma_{L}^{p}$)	$\gamma_{L}^{+}$	$\gamma_{L}^{-}$
Distilled Water	72.8	21.8	51.0	25.5	25.5
Diiodomethane	50.8	50.8	0	0	0

**Table 3:** Relative Concentration of Compositional Atoms and N 1s Functional Groups for BFs samples.

Samples	Composition (At %)			N/C atomic ratio	Ratios of functional group (N 1s) (%)		
	Carbon	Oxygen	Nitrogen		R_1_–NH_2_	R_1_–NH–R_2_	C=NR
C–BFs	72.81	27.19	-	-	-	-	-
ODA–BFs	73.36	26.64	-	-	-	-	-
PDA–BFs	69.98	23.79	6.23	0.089	13.63	79.65	6.72
PDA/ODA–BFs	77.75	16.73	5.52	0.071	7.45	81.08	11.47

**Table 4:** Surface energy calculated by OWRK equation.

Samples	$\gamma_{S}$ (mJ/m^2^)	$\gamma_{S}^{d}$ (mJ/m^2^)	$\gamma_{S}^{p}$ (mJ/m^2^)	$\frac{\gamma_{S}^{d}}{\gamma_{S}^{p}}$
C–BFs	45.57	38.54	7.03	5.48
PDA–BFs	49.96	37.28	12.68	2.94
PDA/ODA–BFs	34.45	32.99	1.46	22.60

**Table 5:** Static Mechanical Properties Statistics of the BFs reinforced PBS Composites.

Label	Tensile strength (MPa)	Tensile modulus (GPa)	Flexural strength (MPa)	Flexural modulus (GPa)	Impact strength (kJ/m^2^)
C–BFs	13.62(1.15) ^a^	0.75(0.18) ^a^	31.38(0.67) ^a^	2.05(0.13) ^a^	10.25(1.11) ^b^
PDA–BFs	15.58(0.69) ^b^	0.79(0.12) ^a^	33.72(0.43) ^b^	2.16(0.09) ^b^	9.02(0.84) ^a^
PDA/ODA–BFs	24.13(0.77) ^c^	1.16(0.08) ^b^	44.09(0.51) ^c^	2.67(0.16) ^c^	15.16(0.63) ^c^

Note: The standard deviations of the test results are recorded in parentheses. The statistical differences (*p*-value < 0.05) are labeled with letters following the parentheses.

## Supplementary Materials

The following are available online at http://www.mdpi.com/2073-4360/10/4/461/s1, Note S1: Surface Free Energy Calculation, Figure S1: SEM observations of: (**a**) C–BFs; and (**b**) PDA/ODA–BFs, Figure S2: Effects of fiber surface modification on water absorption of the composites, Table S1: The calculated water diffusion parameters of BFs/PBS composites.
