# Supplementary material for: Mussel-Inspired Polydopamine as a Green, Efficient, and Stable Platform to Functionalize Bamboo Fiber with Amino-Terminated Alkyl for High Performance Poly(butylene succinate) Composites
Source: Polymers (Basel). 2018 Apr 22;10(4):461. doi: 10.3390/polym10040461 (PMC6415363; doi:10.3390/polym10040461)
Supplement: Supplementary file 1 [file polymers-10-00461-s001.pdf]

polymers

MDPI

Supplementary Materials

Supplementary Methods:

Note S1. Surface Free Energy Calculation

On the basis of Yong's equation, the surface free energies of BF samples were calculated, as shown in Equation 1:

$$\gamma_S = \gamma_L \cos\theta + \gamma_{SL}, \quad (1)$$

where γ_S, γ_L, and γ_{SL} represent the surface tension of the solid, liquid, and solid–liquid interfaces, respectively. θ represents the initial contact angle between the solid (S) and the liquid (L) interfaces.

The OWRK method was used to determine the value of γ_S, as presented in Equation 2:

$$\gamma_L(1 + \cos\theta) = 2\sqrt{\gamma_S^d \gamma_L^d} + 2\sqrt{\gamma_S^p \gamma_L^p}, \quad (2)$$

where γ_S^d and γ_L^d represent the dispersion components on the surface of the solid and liquid interfaces, respectively. γ_S^p and γ_L^p denote the polar components on the surface of the solid and liquid interfaces, respectively.

By combining Equations (1) and (2), Equation (3) is obtained:

$$\frac{\gamma_L(1 + \cos\theta)}{2\sqrt{\gamma_L^d}} = \sqrt{\frac{\gamma_L^p}{\gamma_L^d}} \times \sqrt{\gamma_S^p} + \sqrt{\gamma_S^d}, \quad (3)$$

Therefore, two different liquids, namely deionized water (polar) and diiodomethane (non-polar), were tested in this study to obtain the value of θ. In addition, the surface tension and components of the aforementioned liquids (*i.e.* γ_L, γ_L^d and γ_L^p) can be found in Table 2. According to Equation (3) and the graphing method, if $\sqrt{\frac{\gamma_L^p}{\gamma_L^d}}$ is defined as independent variable (x) and $\frac{\gamma_L(1 + \cos\theta)}{2\sqrt{\gamma_L^d}}$ is defined as (y), a linear relationship is established as shown in Equation 4:

$$y = \sqrt{\gamma_S^p} \cdot x + \sqrt{\gamma_S^d}, \quad (4)$$

allowing the slope and intercept of the line to be used to calculate γ_S^p and γ_S^d. Consequently, the solid surface free energy γ_S can be obtained by Equation 5:

$$\gamma_s = \gamma_S^p + \gamma_S^d, \quad (5)$$

Supplementary Figures and Tables:

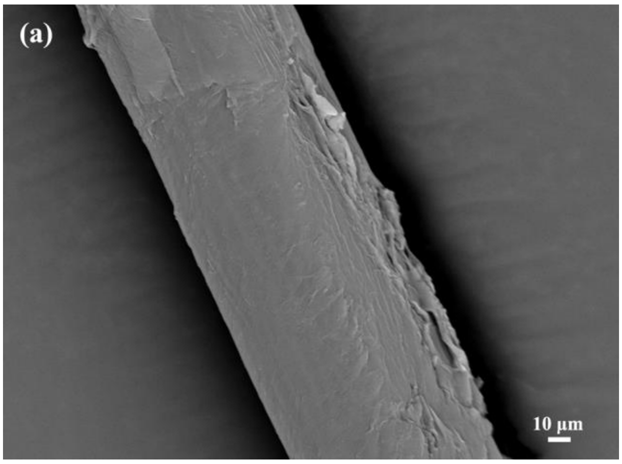

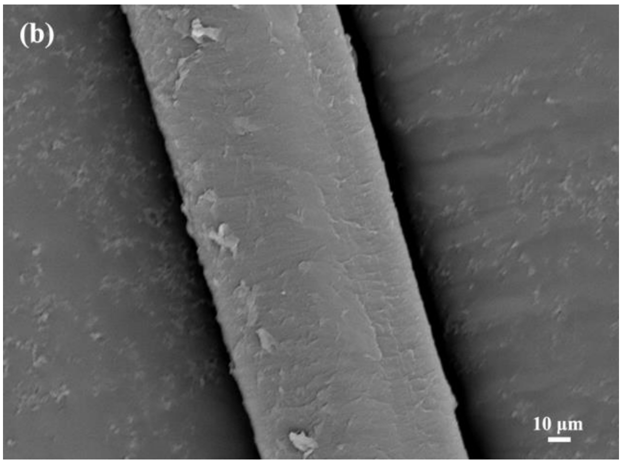

Figure S1. SEM observations of (a) C-BFs and (b) PDA/ODA-BFs

The relatively smooth surface is apparent in the C-BFs sample, which is in accordance with the typical characteristics of natural bamboo fibers (Figure S1a). After coating with polydopamine (PDA) in a mildly conditions just like marine environment, a much rougher film composed of PDA/ODA coating was observed on the fiber surface (Figure S1b). It was reported that the traditional modified method with harsh reaction environment, including hot air and strong alkaline solution, can destroyed the natural structures and components of lignocellulose fibers through decomposing or removing the hemicelluloses and lignin, leaving many small holes and crevices on the surface [1,2]. As expected, after the mussel-inspired surface modification pathway, the distinct structure and components of BF were retained, because the whole process of PDA decoration was conducted under mail conditions, avoiding causing the aforementioned damages.

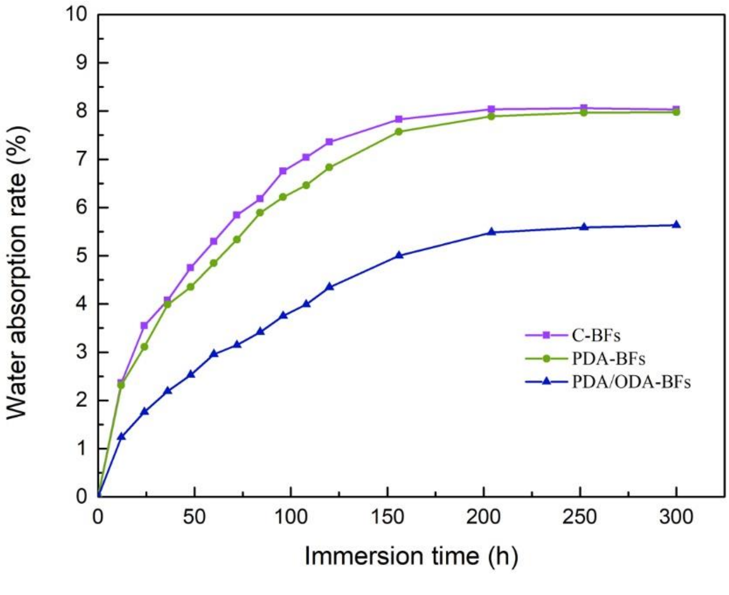

Figure S2. Effects of fiber surface modification on water absorption of the composites.

Table S1. The calculated water diffusion parameters of BFs/PBS composites

**Label**	***n***	***k*** **($h^{-1/2}$)**	***R*²**	**M_e[1] (%)**	**D[2]×10^{-13} (m^2/s)**
C-BFs	0.4743	0.0942	0.9943	8.03±0.58	1.20
PDA-BFs	0.4745	0.0885	0.9971	7.97±0.42	1.08
PDA/ODA-BFs	0.5397	0.0566	0.9982	5.63±0.14	0.79

[1] M_e represents the equilibrium moisture content of the composites; [2] D represents the diffusion coefficient.

References

1. Wang, F.; Zhou, S.; Yang, M.; Chen, Z.; Ran, S. Thermo-Mechanical Performance of Polylactide Composites Reinforced with Alkali-Treated Bamboo Fibers. *Polymers* **2018**, *10*, 401.
2. Cheng, H.T.; Gao, J.; Wang, G.; Shi, S.Q.; Zhang, S.B.; Cai, L.P. Enhancement of mechanical properties of composites made of calcium carbonate modified bamboo fibers and polypropylene. *Holzforschung* 2015, 69, 215-221.

Article Information

Title: Mussel-Inspired Polydopamine as a Green, Efficient, and Stable Platform to Functionalize Bamboo Fiber with Amino-terminated Alkyl for High Performance Poly(butylene succinate) Composites

Author Contributions: Gonghua Hong, Shuangbao Zhang, and Wei Song conceived the project and designed the experiments; Gonghua Hong wrote the main manuscript text; Gonghua Hong, Yang Meng, Jianyong Lin, Zhenghao Chen, and Wei Song performed the experiments and analyzed the data; Shuangbao Zhang, Wei Song, and Haitao Cheng supervised and directed the project; all authors reviewed the manuscript.

Acknowledgments: This work was supported by the National Natural Science Foundation of China (31670571), Beijing Natural Science Foundation (6162019), National Key R & D Program of China (2017YFD0600804), and Co-built Foundation with Zhejiang Province "R & D on Natural Fiber Composites and Environmentally Friendly Adhesives" (CZXC201410).
